# Biomechanical Test Following Removal of a Dynamic Hip Screw: In Vitro Analysis

**DOI:** 10.7759/cureus.3680

**Published:** 2018-12-04

**Authors:** Anderson Freitas, Jhefferson B Breta, Joubert Júnior, Antônio C Shimano, Walter R Daher, Munir Bessa, Weverton P De Alcantara, Lucas Sacramento Ramos, Ergon L Dantas, Ruben J Aquino

**Affiliations:** 1 Orthopaedics, Hospital of Orthopedic and Specialized Medicine, Brasilia, BRA; 2 Orthopedics and Traumatology, Hospital Regional Do Gama, Brasilia, BRA; 3 Orthopaedics, Hospital Regional Do Gama, Brasília, USA; 4 Orthopaedics, Universidade de São Paulo, São Paulo, BRA; 5 Orthopedics and Traumatology, Hospital Regional De Ceilândia, Brasilia, BRA

**Keywords:** femoral fractures, fracture fixation, internal, osteoporosis

## Abstract

The objective of this study was to evaluate, by means of a static flexural test, the biomechanical parameters necessary for the occurrence of a proximal femoral fracture in a synthetic bone model after the removal of a dynamic hip screw (DHS) and comparing the results obtained with a reinforcement technique using polymethylmethacrylate (PMMA).

Twenty synthetic bones made of the same material and from the same manufacturer were used: ten units as the control group (CG), five units as the test group without reinforcement (TG), and five units as the test group with reinforcement (TGR). The biomechanical analysis was performed simulating a fall over the trochanter using a servo-hydraulic machine. In the control group, the assay was performed with its integrity preserved. In the TG and TGR groups, a DHS model was introduced, and the tests were performed as follows: TG after simple removal of the synthesis material, and in the TGR group, after removal of the synthesis material and filling the orifice of the femoral neck with PMMA.

All groups presented with a basicervical fracture of the femoral neck. The CG group presented a mean of 935 newtons (N) of maximum load and 7.0 joules (J) of energy for fracture occurrence. TG and TGR groups presented, respectively, a maximum load of 750 N and 1,068 N, and energy of 6.0 J and 7.3 J. According to the one-way analysis of variance (ANOVA), there was no significant difference in flow load (p = 0.16), energy to flow (p = 0.16), stiffness (p = 0.28), maximum load (p = 0.10), and energy to fracture (p = 0.54) between the studied groups.

The removal of the DHS implant from the synthetic bone did not present a significant increase of the maximum load and the energy necessary for the occurrence of a fracture with the use of the PMMA reinforcement technique.

## Introduction

Osteoporosis is a public health problem of the world population and a disease that is most prevalent in the elderly female population. This disease is characterized by a reduction of the bone mineral density and thus, a reduction of the mechanical resistance of this tissue. It has, as its main socioeconomic impact, the occurrence of fractures by low energy trauma; of these, the fracture of the proximal end of the femur is the one with the highest mortality rates [1].

The aim of treating these fractures is to allow the resumption of the patient’s daily activities as quickly as possible, either by joint replacement (hip arthroplasty) or by fracture osteosyntheses, such as a proximal femoral nail (PFN), cannulated screws, or dynamic hip screw (DHS) [2].

The indications for withdrawal of the synthesis used for a fracture treatment of the proximal femoral end are persistent pain in the gluteal and thigh region, which may be caused by the prominence of the synthetic material (DHS, cannulated screw, or stem), implant failure, or infection [2-3]. After the consolidation of the proximal femoral fracture, removal of implants may cause complications, such as possible fractures of the femoral neck or in the intertrochanteric region, especially in patients with poor bone quality [3-5].

Thus, describing the results by means of a static flexion test simulating a fall over the great trochanter in synthetic bones after removal of a material synthesis (DHS), with and without a reinforcement technique, will allow us to describe the mechanical behavior of this region and determine the real concern in removing this material.

## Materials and methods

The aim of this study was to evaluate the resistance and energy required for the occurrence of a proximal femoral fracture in the synthetic bone after removal of the DHS implants and comparing the results obtained with the bone cement reinforcement technique.

Twenty synthetic femurs (Model c1010) from a supplier in Brazil (Nacional Ossos®, Jaú-Sao Paulo, Brazil), made of sponge and cortical polyurethane with 10 PCF (pounds per cubic foot) and a 12 mm medullary canal, were used in this evaluation. Those 20 synthetic femurs (of the same batch and model number) were divided into three groups: a control group (CG), a test group without reinforcement (TG), and a test group with reinforcement (TGR), composed of 10, five, and five units per group, respectively.

**Figure 1:**
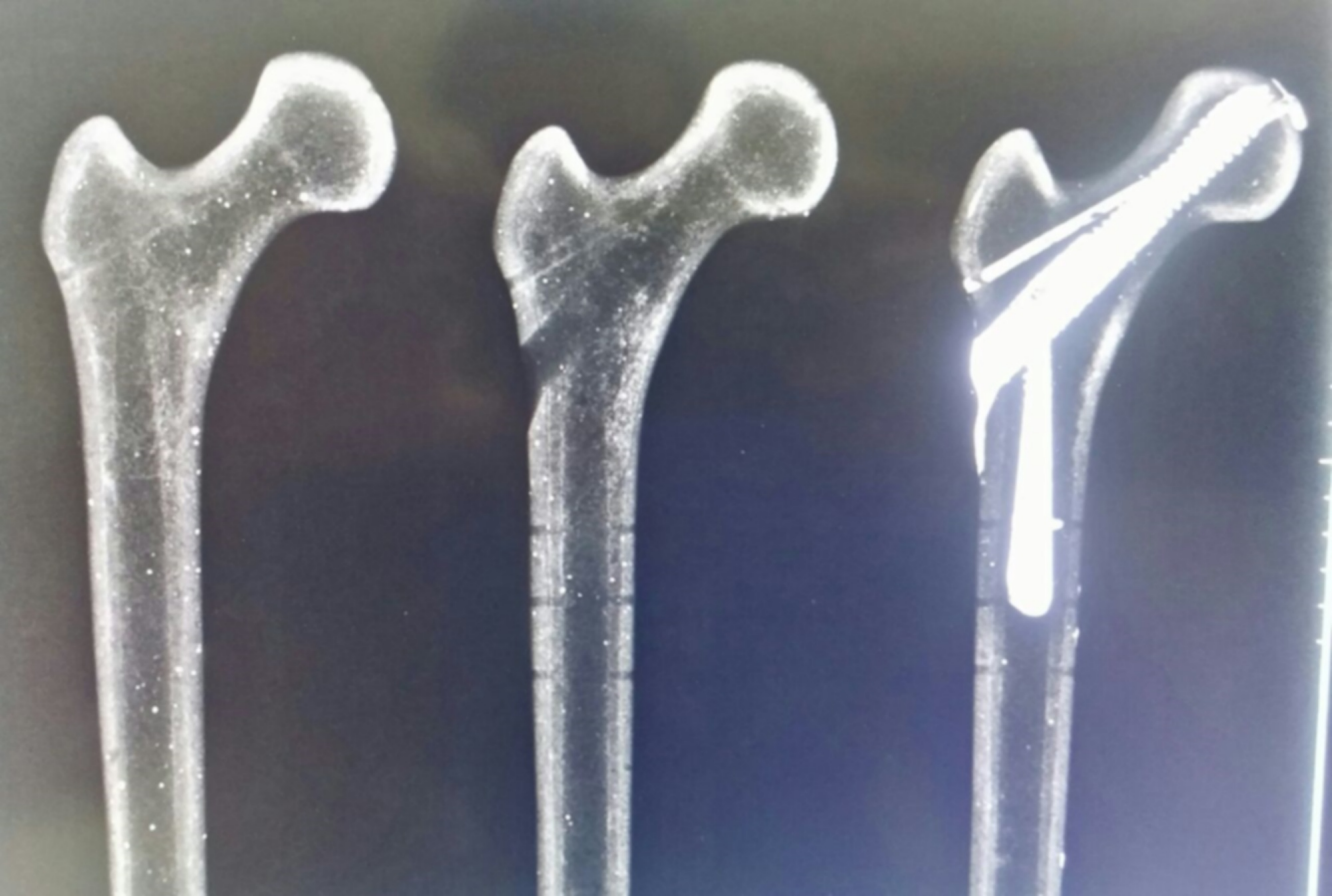
Radiographic image of the synthetic models CG, TG, and TGR, respectively, before the biomechanical test CG: control group; TG: test group without reinforcement; TGR: test group with reinforcement

The CG was formed by synthetic femurs, internally and externally intact and without previous fractures. In the TG and TGR groups, the synthetic femurs were submitted for DHS implantation using a 12 mm diameter / 95 mm long sliding screw, according to Baumgartner's tip apex distance (TAD) of up to 25 mm, assisted by radioscopy in all specimens and withdrawn soon thereafter. In the TG group, a biomechanical test was performed shortly after removing the implant without any reinforcement technique. In the TGR group, after the implant was removed, the synthetic femurs were submitted to a reinforcement technique with polymethylmethacrylate (PMMA) in the sliding screw orifice, introduced anterograde with the assistance of a 20 ml syringe, using 10.7 ml (on average) to fill the space (Figure 1). Normal viscosity PMMA was purchased from Biomecânica® (Jaú-Sao Paulo, Brazil).

The bone models that lost cement to the diaphysis beyond the smaller trochanter were excluded from the study (due to the possibility of altering the biomechanics of the proximal femur) since some specimens had an inadvertent filling of part of the femoral canal, causing lack of PMMA in the femoral head.

All samples were sent to the biomechanical testing laboratory. The servo hydraulics MTS 810 Flex Test 40 (MTS Sistemas do Brasil Ltda., São Paulo-SP, Brazil) with a capacity of 100 kN was used statically in flexion.

**Figure 2:**
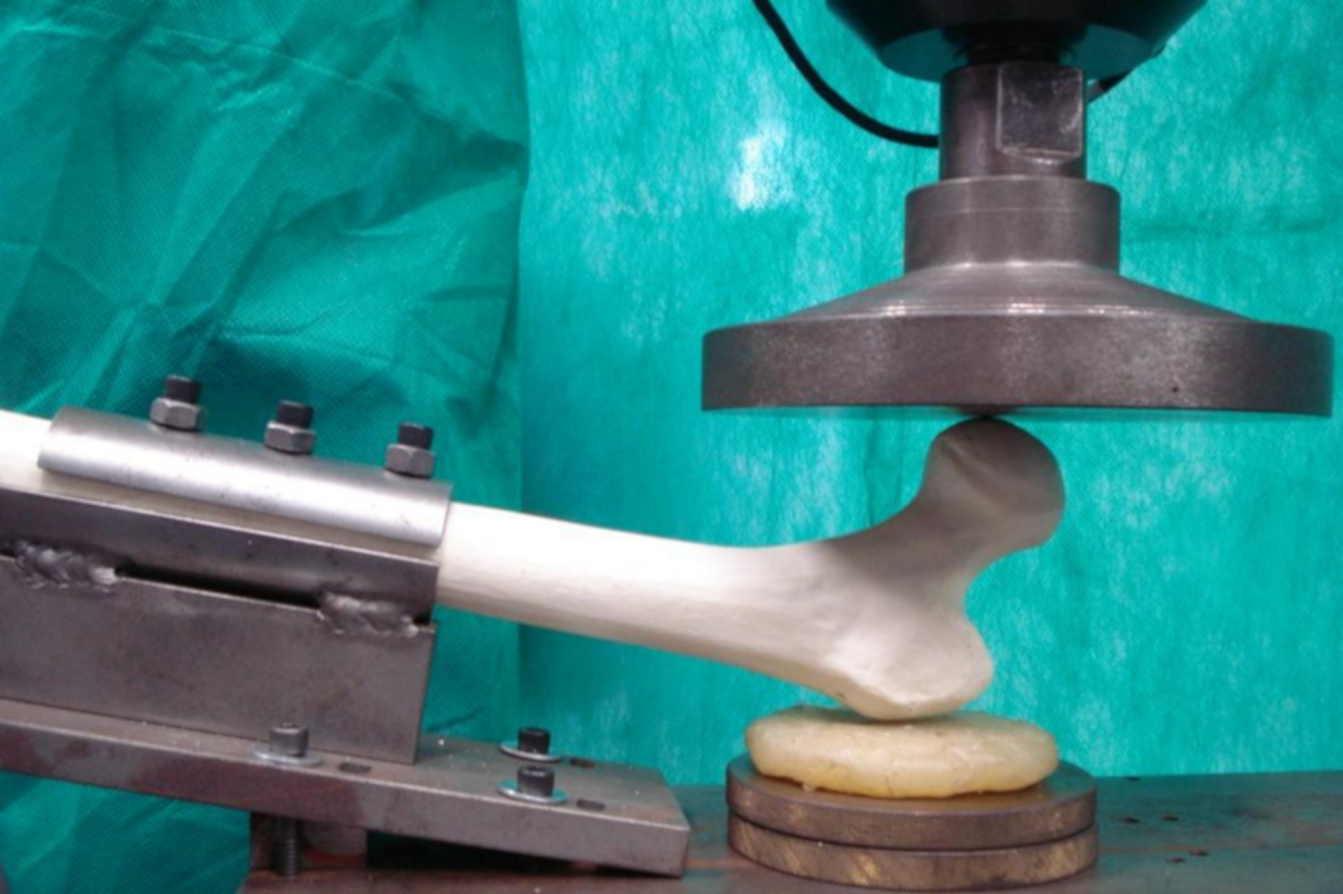
Femur adjusted on the device during test

**Figure 3:**
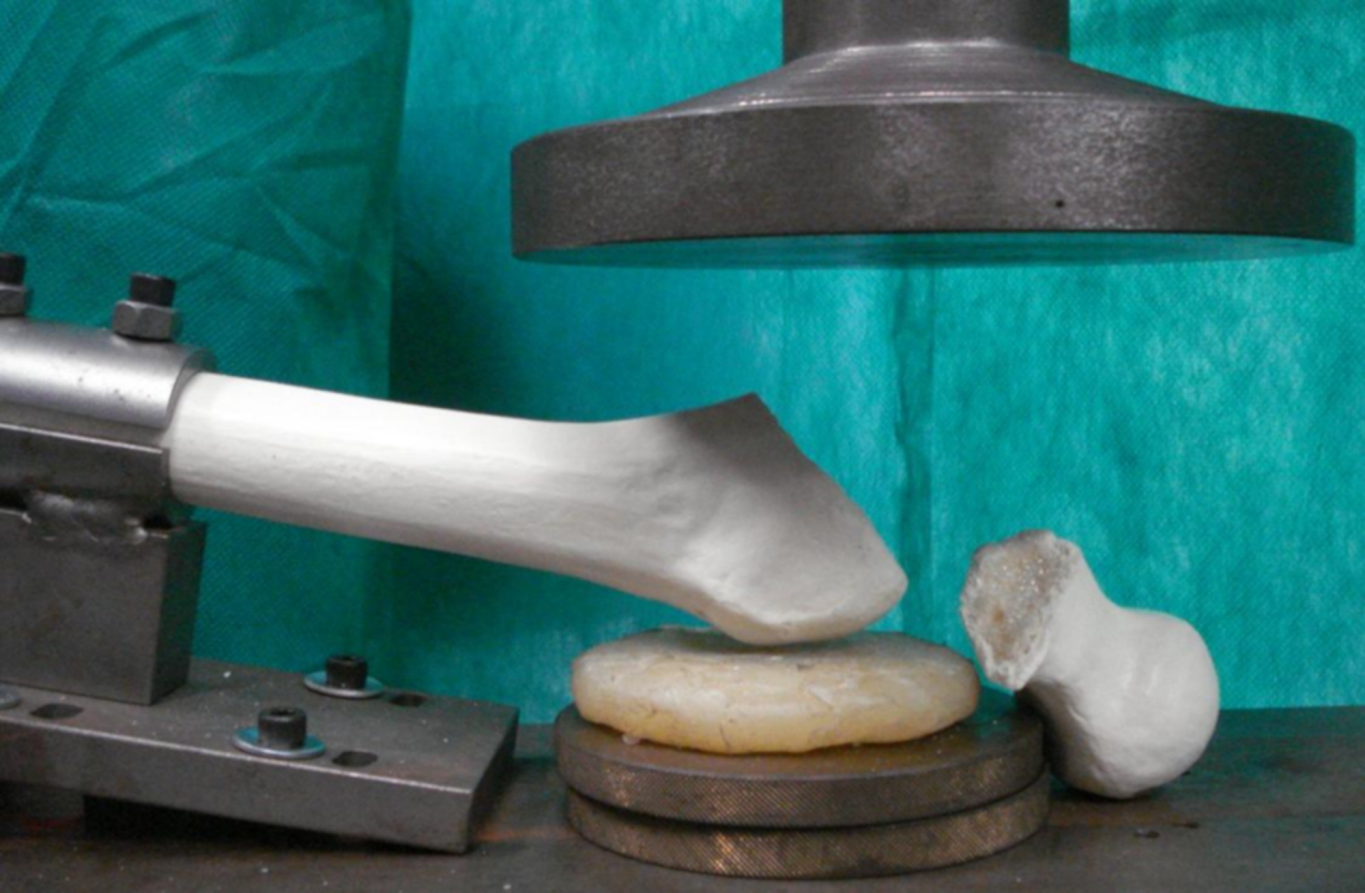
Femur after the test

The femur was attached to the test device leaving 150 mm of its length out of the device towards the hydraulic piston, positioned at the base of the test machine at 10° inclination with the horizontal and 15° internal rotation measured with a digital goniometer. The greater trochanter was kept resting on an 8 x 2 cm diameter silicone disc (Figures 2-3).

A 40 N preload was applied at 2 mm/s and then the load was applied to the femur head to reach fracture, obtaining the values of maximum load in newton (N) and energy in joule (J).

The results were obtained through an inferential analysis using the values of the selected parameters, composed by the analysis of variance (ANOVA) one-way, with the objective of verifying whether there was a significant difference of the values obtained between CG, TG, and TGR. The significance level was 5%. The statistical analysis was performed by the IBM Statistical Package for Social Sciences (SPSS) Statistics, version 20 (IBM SPSS Statistics, Armonk, NY).

## Results

**Figure 4:**
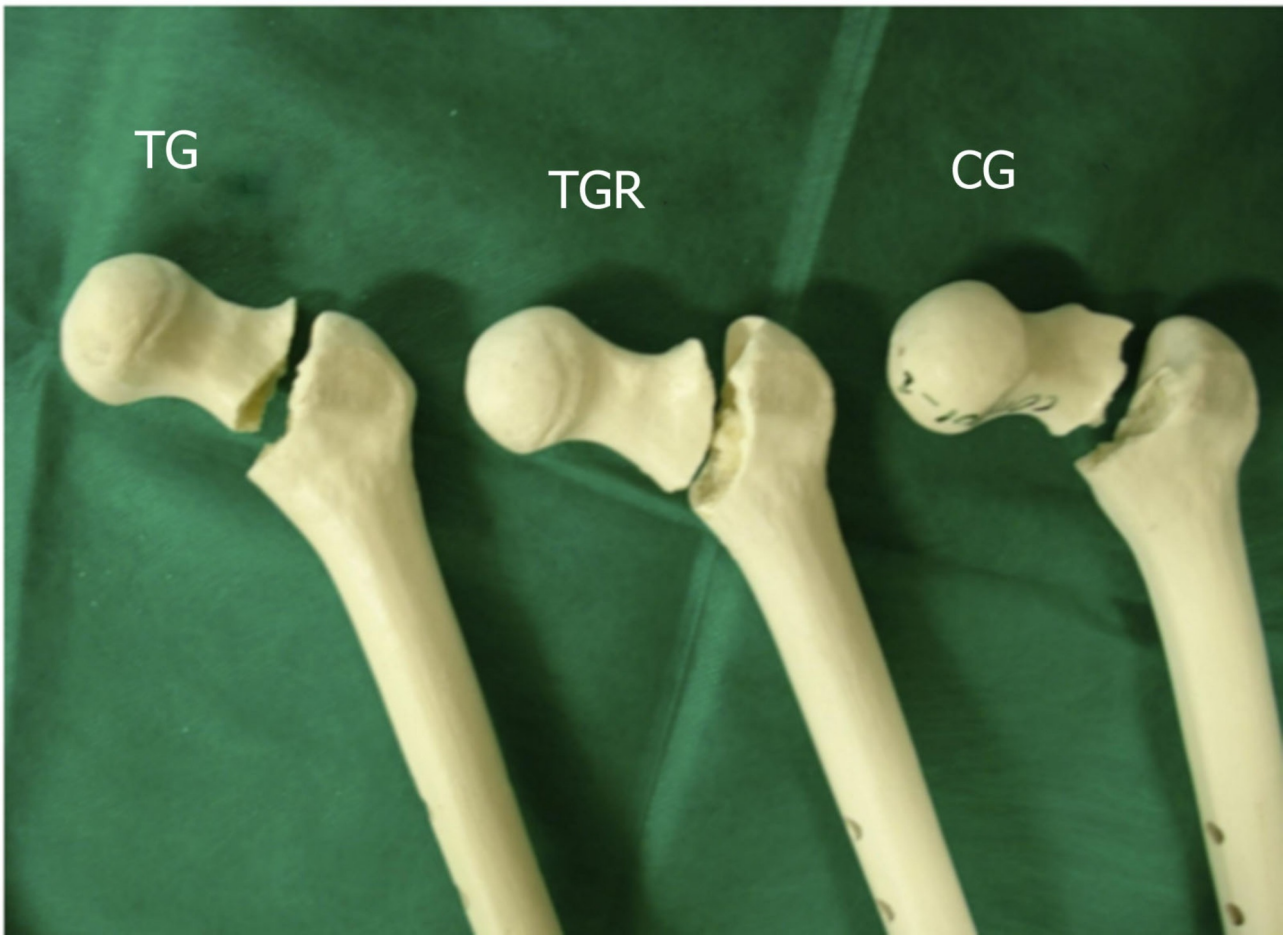
All specimens tested showed a basicervical fracture CG: control group; TG: test group without reinforcement; TGR: test group with reinforcement

All specimens tested showed a basicervical fracture (Figure 4). The mean PMMA value used to fill the orifices of the DHS slide bolts of the TGR model was 11 ml.

The parameters analyzed in the GC, TG, and TGR groups presented the following averages, respectively: flow load in N (newton): 697, 565, and 808; energy to flow in J (joule): 2.76, 2.08, and 3.32; stiffness in N/mm: 90, 79, and 102; maximum load in N: 935, 750, and 1,068; and energy to reach fracture in J: 7.1, 6.0, and 7.3 (Tables 1-2).

**Table 1:** Flow and Stiffness Parameters for the Control and Test Groups *analysis of variance (ANOVA) one-way CG: control group; CI: confidence interval; J: joule; N: newton; TG: test group without polymethylmethacrylate (PMMA) reinforcement; TGR: test group with PMMA reinforcement

Variable	n	mean	CI 95% for the mean		minimum	maximum	p-value*		
Flow load (N)									
CG	10	697	559	835	316	1,010			
TG	5	565	518	612	509	630	0.16		
TGR	5	808	628	989	592	1,022			
Displacement to flow (mm)									
CG	10	7.71	7,0	8.4	5.3	9.5			
TG	5	7.26	6.2	8.4	5.4	8.7	0.70		
TGR	5	7.29	6.5	9.4	5.3	9.5			
Energy to flow (J)									
CG	10	2.76	2.2	3.4	0.9	3.8			
TG	5	2.08	1.7	2.5	1.4	2.7	0.16		
TGR	5	3.32	2.1	4.5	1.7	4.8			
Stiffness (N/mm)									
CG	10	90	73	107	60	138			
TG	5	79	9	89	64	94	0.28		
TGR	5	102	88	117	77	118			

**Table 2:** Maximum Load and Energy Parameters Necessary to Reach Fracture for the Control and Test Groups CG: control group; CI: confidence interval; J: joule; N: newton; TG: test group without polymethylmethacrylate (PMMA) reinforcement; TGR: test group with PMMA reinforcement

Variable	n	mean	CI 95% for the mean		minimum	maximum	p-value*			
Flow load (N)										
CG	10	935	755	1115	555	1399				
TG	5	750	689	812	663	829	0.10			
TGR	5	1068	963	1172	870	1162				
Energy to reach fracture (J)										
CG	10	7.1	5.5	8.6	4.4	10.4				
TG	5	6.0	4.9	7.1	4.0	7.0	0.54			
TGR	5	7.3	6.2	8.4	6.3	9.0				
CG: control group; TG: test group without PMMA reinforcement; TGR: test group with PMMA reinforcement; *ANOVA one-way										

According to the one-way ANOVA, there was no significant difference in flow load (p = 0.16), energy to flow (p = 0.16), stiffness (p = 0.28), maximum load (p = 0.10), and energy to reach fracture (p = 0.54) between the groups analyzed.

## Discussion

Removal of implants with considerable diameters, such as the DHS after fracture healing, may bring up considerable concerns, especially in osteoporotic bone, so it is important to develop studies that describe the behavior of this region after the withdrawal of the synthesis [5].

The use of bone reinforcement (PMMA) after implant withdrawal has already been studied, although there is a concern regarding the amount used due to the local thermal reaction [6-7]. However, the average volume we reached approaches other studies that demonstrate little local thermal variation.

The choice of synthetic bones was to ensure comparable biomechanical properties between the groups and to eliminate variables found in cadaveric models (bone density, length, and diameter) [8]. As seen in the study by Paiva et al., we noticed that there was a large difference interval in the control group; therefore, we chose to increase the number of synthetic bones to 10 in the control group in order to improve this interval [9].

Although the absolute values are not comparable to studies presented in the literature that used cadaveric bones, the behavior of the increase of load in the TGR and TG groups reduction when compared to the CG allowed us to observe a regular behavior among the trials, even if there was no statistical variance [10-11].

We compared our results to a similar work in which the authors used the same method and synthetic model but using a proximal femoral nail (PFN) instead of a DHS [9]. Paiva et al. described statistically significant results comparing the groups with and without cement filling, a very intriguing result, since the PFN screw used in the study was 10.5 mm in diameter, smaller than the 12.0 mm of the DHS screw used in our research. We also expected that using a DHS would lead to the non-invasion of spongy bone in the region of the greater trochanter and that could provide greater resistance as compared to the study using a PFN, although we were not able to confirm these expectations.

Nevertheless, we noticed a change that might have caused the difference. The proximity of the cement filling to the uppermost part of the femoral neck in the PFN group, as compared to our study with DHS, was on the fall over the trochanter. It is, therefore, possible that the closer to this region, the greater the resistance implemented by the reinforcement, leading us to believe that one of the main factors of the increased strength with PMMA in the proximal femoral extremity is not only about the volume of PMMA used but also its positioning, a fact already mentioned in other publications [9-12].

The TG group did not show a significant difference as compared to the CG, leading us to believe that the protection provided by filling the sliding bolt orifice after removing the DHS can be excessive, possibly making it difficult to make a synthesis, in case of a fracture after its withdrawal in the presence of PMMA.

## Conclusions

The experiments of the present study did not show a significant difference between the studied groups; further clinical developments and research are needed to ratify the results presented.
